# The Control of Zoonotic Soil-Transmitted Helminthoses Using Saprophytic Fungi

**DOI:** 10.3390/pathogens9121071

**Published:** 2020-12-21

**Authors:** Cándido Viña, María Isabel Silva, Antonio Miguel Palomero, Mathilde Voinot, María Vilá, José Ángel Hernández, Adolfo Paz-Silva, Rita Sánchez-Andrade, Cristiana Filipa Cazapal-Monteiro, María Sol Arias

**Affiliations:** Control of Parasites Research Group (COPAR, GI-2120), Department of Animal Pathology, Faculty of Veterinary, University of Santiago de Compostela, 27142 Lugo, Spain; candidovina@hotmail.es (C.V.); ma.isabel.st@hotmail.com (M.I.S.); dim772@hotmail.es (A.M.P.); mat.vmei@gmail.com (M.V.); maria.vila.pena@gmail.com (M.V.); joseangelher@gmail.com (J.Á.H.); rita.sanchez-andrade@usc.es (R.S.-A.); cristianafcm@gmail.com (C.F.C.-M.); mariasol.arias@usc.es (M.S.A.)

**Keywords:** soil-transmitted helminths, zoonoses, parasiticide fungi, one health

## Abstract

Soil-transmitted helminths (STHs) are parasites transmitted through contact with soil contaminated with their infective eggs/larvae. People are infected by exposure to human-specific species or animal species (zoonotic agents). Fecal samples containing eggs of *Ascaris suum* or *Lemurostrongylus* sp. were sprayed with spores of the soil saprophytic filamentous fungi *Clonostachys rosea* (CR) and *Trichoderma atrobrunneum* (TA). The antagonistic effect was assessed by estimating the viability of eggs and their developmental rate. Compared to the controls (unexposed to fungi), the viability of the eggs of *A. suum* was halved in CR and decreased by two thirds in TA, while the viability of the eggs of *Lemurostrongylus* sp. was reduced by one quarter and one third in CR and TA treatments, respectively. The Soil Contamination Index (SCI), defined as the viable eggs that attained the infective stage, reached the highest percentages for *A. suum* in the controls after four weeks (66%), with 21% in CL and 11% in TA. For *Lemurostrongylus* sp., the values were 80%, 49%, and 41% for control, CR and TA treatments, respectively. We concluded that spreading spores of *C. rosea* or *T. atrobrunneum* directly onto the feces of animal species represents a sustainable approach under a One Health context to potentially reduce the risk of zoonotic STHs in humans.

## 1. Introduction

Soil-transmitted helminths (STHs) are parasites characterized by adults that excrete eggs in infected individuals, which are then passed in the feces to the soil, where they develop into the infective stages [1]. For roundworms and whipworms, infection occurs when eggs containing larva are ingested [2], whereas the eggs of Strongylids/hookworms that hatch into first-stage larva can emerge and molt into a third-stage larva (L3, infective phase), which moves towards the hosts to penetrate their skin or be accidentally ingested [3].

These parasites provoke around 80% of helminth infections in humans, with the most important etiological agents being the roundworm *Ascaris lumbricoides*, the whipworm *Trichuris trichiura*, and the hookworms *Necator americanus*, *Ancylostoma duodenale*, and *Ancylostoma ceylanicum* [4]. Significant morbidity is observed in hundreds of millions of infected people, with children displaying the greatest values. These diseases are considered neglected infections by the WHO, which estimates that about one and a half billion people are infected, especially when exposed to risk factors such as inappropriate water supply and sanitation, crowded living conditions, a lack of access to health care, and low levels of education [5,6]. The risk of human infection by STHs can worsen as roundworms of animals (*Toxocara canis*, *Ascaris suum*, *Baylisascaris procyonis*), whipworms (*Trichuris* spp.), and hookworms (*Ancylostoma caninum*) have zoonotic potential [7]. For example, children owning pets and/or enjoying areas with exposed earth, such as gardens, parks, or sandpits, where dogs are frequently observed are at elevated risk of infection by *T. canis* and/or *A. caninum*. Infection by *B. procyonis* among children is related to the presence of feces from infected raccoons in the environment surrounding people’s homes [8,9,10].

Because the transmission of STHs is closely associated with soil contamination according to infective stages, several environmental actions have been considered to reduce the risk of livestock infection [11]. Certain saprophytic filamentous fungi such as *Duddingtonia flagrans*, *Monacrosporium thaumasium*, and *Arthrobotrys* spp. are nematophagous species able to trap larvae of Strongylids/hookworms [12,13]; other species (*Mucor circinelloides*, *Pochonia chlamydosporia*, *Trichoderma* spp., and *Purpureocillium lilacinum*) can penetrate the eggshells of trematodes, roundworms, and whipworms to access their inner portions [14,15,16]. Therefore, in order to control both infective eggs or larvae a blend of fungi with complementary activities, i.e., ovicide and larvicide activities, should be spread [17].

Since some of the STHs affecting humans are caused by infective stages originating from eggs shed in the feces of infected animals, this problem must be treated under a One Health approach, which involves providing a unique solution for reducing the levels of soil contamination by disrupting the lifecycles of the parasites in the soil [18]. Accordingly, the possibility of lessening the viability of eggs of the roundworm *A. suum* and the Strongylid *Lemurostrongylus* sp., together with their ability to attain infectivity, was tested in the present study by spraying spores of *Clonostachys rosea* and *Trichoderma atrobrunneum* directly onto the feces of pigs and lemurs.

## 2. Results

### 2.1. Recovery Rates of Eggs of STHs

Using flotation test, we observed that the *A. suum* egg-output values oscillated between 525 ± 155 eggs per gram of feces (EPG) in TA (exposed to *T. atrobrunneum*) and 625 ± 290 in CR (exposed to *C. rosea*) at the start of the trial (Table 1). The recovery rates varied among the experiments, with the lowest numbers observed at the fourth week (51% in the control, 42% in CR, and 38% in TA). Significant differences were demonstrated between the control and CR and between the control and TA (Z = −2.795, *p* = 0.005 and Z = −2.2655, *p* = 0.006, respectively).

Fecal counts of eggs of *Lemurostrongylus* sp., ranging from 490 ± 188 EPG in the control to 750 ± 177 EPG in CR, were detected at the beginning of the trial (Table 1). No eggs were observed in the control from the second week (recovery rate 67%), whereas in the third week, the lowest percentage of eggs was recovered in CR (41%) and TA (43%). The differences between the control and CR and between the control and TA were significant (Z = −3.414, *p* = 0.001 and Z = −2.043, *p* = 0.029, respectively).

### 2.2. Viability of Eggs of STHs

#### 2.2.1. *Ascaris suum*

At the beginning of the study, the percentage of viability was 99% (Table 2), which reduced in the control to 81% after four weeks (wk). In the samples featuring added spores of *C. rosea* (CR), a significant decrease was recorded throughout the study, with the lowest values observed in the fourth week (34%). Spraying spores of *T. atrobrunneum* (TA) on the swine fecal samples decreased the viability of the eggs of *A. suum* to 28%. Significant differences were demonstrated between the control and CR and between the control and TA (Z = −3.382, *p* = 0.001 and Z = −3.247, *p* = 0.001, respectively). No differences were recorded between CR and TA (Z = 0.892, *p* = 0.904).

#### 2.2.2. *Lemurostrongylus* sp.

The percentage of viability of the eggs of *Lemurostrongylus* sp. was 99% at week 0 (Table 3). In the control, the viability decreased to 90% until the second week, at which point eggs were not observed. After adding spores of *C. rosea*, percentages of viability around 63% were observed in CR (third week). In TA, the viability lessened by 64% by the third week of the assay. The differences between the control and CR and TA were significant (Z = −2.765, *p* = 0.006 and Z = −2.649, *p* = 0.008, respectively). No differences were observed between CR and TA (Z = −0.495, *p* = 0.621).

### 2.3. Development of Eggs of STHs

#### 2.3.1. *Ascaris suum*

**Figure 1:**
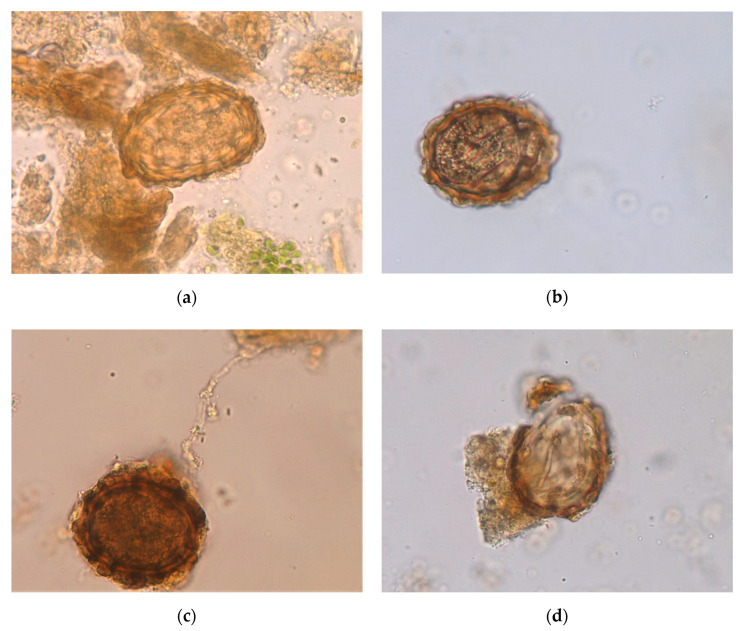
Development of eggs of *A. suum* in the feces of pigs. (**a**) Unembryonated egg (zygote). (**b**) Infective egg containing an L2 larva inside. (**c**) The development of eggs was delayed in the presence of *Clonostachys rosea*. (**d**) Destruction after exposure to *Trichoderma atrobrunneum*.

All the viable eggs passed in the feces were in the zygote stage at the beginning of the study (Table 2, Figure 1). In the eggs unexposed to fungi (control), notable cellular division was noted in the first week (71%), followed by the appearance of eggs containing an L1 inside (second week) (70%) and later an L2 (third week) (59%), reaching the highest numbers by the fourth week (85%).

In CR, a similar pattern was observed, although the development of the eggs was slower than that in the control (Table 2). During the first week, 63% of eggs showed developing embryos, 49% showed an L1 by the second week, and 31% contained an L2 by the third week. The percentage of infective eggs at the end of the study was 59%.

Half of the eggs in TA remained at the zygote stage one week after the addition of spores of *T. atrobrunneum*, and 39% remained at this stage by the second week (Table 2); a total of 24% of eggs had an L1 by the third week, while the percentages of eggs with an L2 was 39% one week later.

Significant differences were obtained for the eggs with a zygote between the control and CR (U = −1.983, *p* = 0.049) and between the control and TA (U = −2.689, *p* = 0.007). No differences were observed among the eggs treated with the two filamentous fungi.

#### 2.3.2. *Lemurostrongylus* sp.

**Figure 2:**
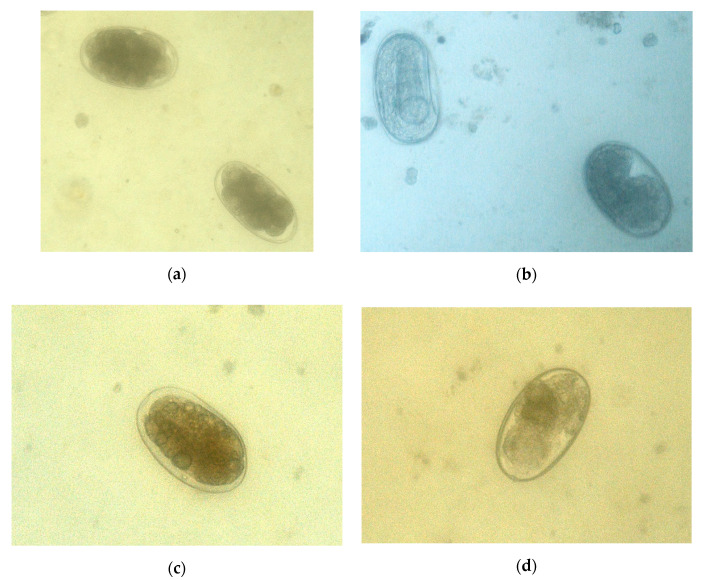
Development of eggs of *Lemurostrongylus* sp. in the feces of lemurs. (**a**) Eggs in the phase of developing embryos. (**b**) First larvae appeared from the 1st week. (**c**) The exposure to *Clonostachys rosea* caused irreversible damage through vacuolization. (**d**) In the presence of *Trichoderma atrobrunneum*, the inner content was destroyed.

As summarized in Table 3, ninety-one percent of the eggs of *Lemurostrongylus* sp. were in the zygote stage at the beginning of the study (Figure 2). Rapid development was noted in the eggs unexposed to fungi (control), with 77% reaching the L1 stage in the first week and 90% in the second week. No eggs with cellular division or L1 within were observed after this time.

In CR, 45% of eggs reached the L1 stage in the first week, whereas this percentage was 74% in the third week (Table 2) (Figure 2). One week after the addition of spores of *T. atrobrunneum* (TA), 26% of the eggs had an L1 inside, with 51% by the third week.

Significant differences were obtained between the control and CR regarding the eggs with zygotes (U = −3.842, *p* = 0.001), developing embryos (U = −4.056, *p* = 0.001), and L1 (U = −3.610, *p* = 0.001); differences between the control and TA were found in the eggs with zygotes (U = −3.881, *p* = 0.001), developing embryos (U = −4.405, *p* = 0.001), and L1 (U = −3.842, *p* = 0.001). No differences were demonstrated among the eggs treated with the two filamentous fungi.

### 2.4. Effect of SSF (Soil Saprophytic Fungi) on the Risk of Infection by Eggs and Larvae of STHs

**Figure 3:**
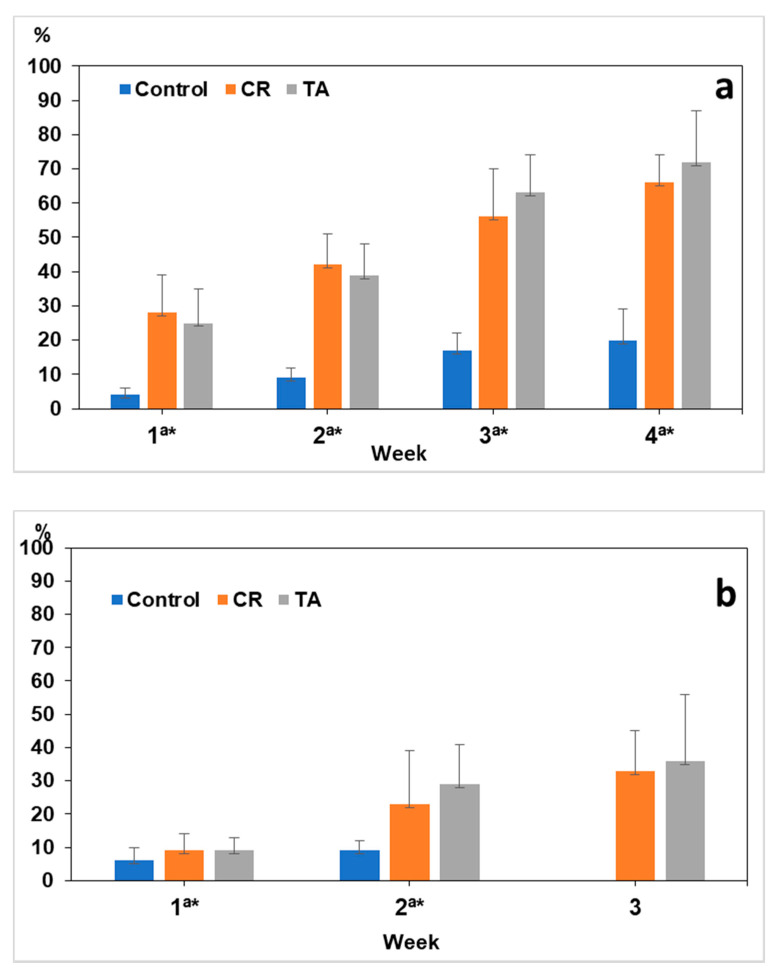
Values of the Viability Reduction (VR) of eggs of *A. suum* (**a**) and *Lemurostrongylus* sp. (**b**). Control: not exposed to fungi; CR: exposed to the parasiticide fungus *Clonostachys rosea*; TA: exposed to *Trichoderma atrobrunneum*. Bars are 95% Confidence Interval. a: Significant differences between Control and CR; *: significant differences between Control and TA.

As illustrated in Figure 3, the values of viability reduction (VR) varied from 4% to 20% in the eggs of *A. suum* not exposed to SSF. An important increase in the VR was observed in CR and TA, which presented percentages higher than 60% after four weeks.

For the eggs of *Lemurostrongylus* sp., the control values of VR oscillated between 5% (first week) and 9% (second week), whereas those in CR ranged from 10% to 33%, and those in TA ranged from 9% to 36%.

**Figure 4:**
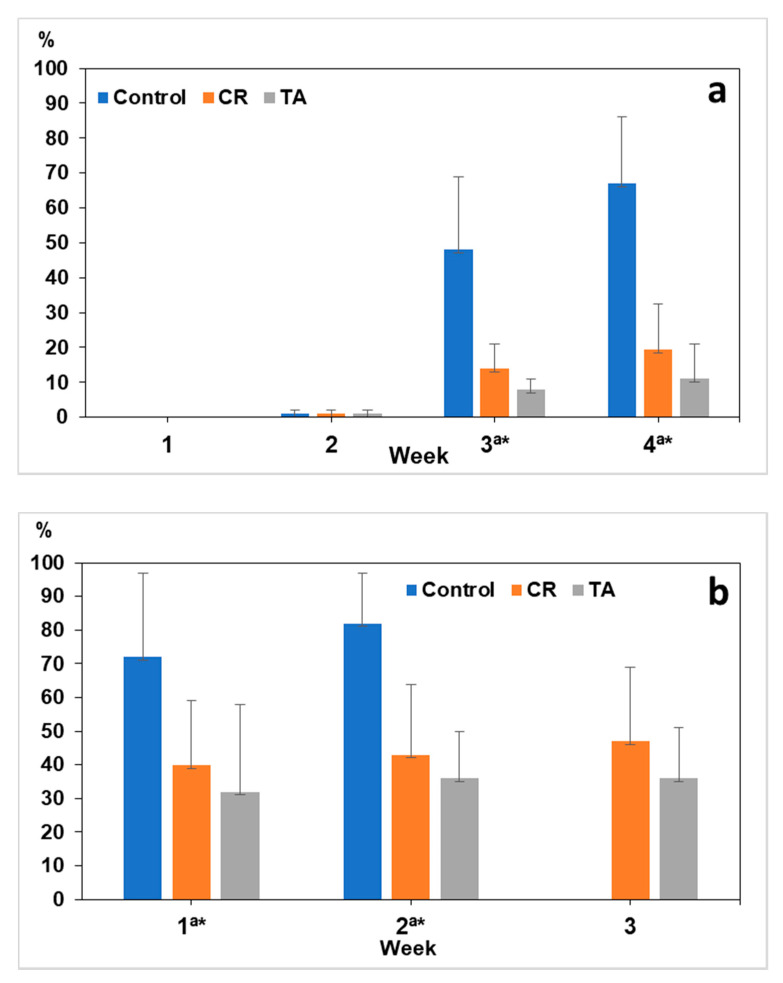
Values of the Soil Contamination Index (SCI) by eggs of *A. suum* (**a**) and *Lemurostrongylus* sp. (**b**). Control: not exposed to fungi; CR: exposed to the parasiticide fungus *Clonostachys rosea*; TA: exposed to *Trichoderma atrobrunneum*. Bars are 95% Confidence Interval. a: significant differences between Control and CR; * significant differences between Control and TA.

The values of the Soil Contamination Index (SCI) increased throughout the study (Figure 4). Regarding contamination of *A. suum* by eggs, the highest percentages were observed in the control after four weeks (67%), by 20% in CR and by 11% in TA.

For *Lemurostrongylus* sp., an SCI higher than 70% was observed in the control from the first week, whereas a value of 47% was obtained in CR after three weeks, and a value of 36% in TA was observed during the same week.

## 3. Discussion

Certain STHs affecting animal species can also infect humans. Because the infective stages of these pathogens originate in the soil from eggs passed into the feces of animal species, the health of people, animals, and soil is of concern [19]. To reduce the levels of ground contamination and thus the possibility of people becoming infected, a study was carried out to determine the possibility of destroying the eggs of a roundworm and a Strongylid, making them permanently unviable, or delaying their development. By spraying the spores of two soil filamentous fungi, *C. rosea* and *T. atrobrunneum*, directly onto the feces of pigs infected by the roundworm *A. suum*, the viability of the eggs dropped by one half and two thirds, respectively, compared to that observed in absence of fungi. When the spores were spread onto the feces of lemurs passing eggs of *Lemurostrongylus* sp., this viability decreased by one quarter three weeks after adding *C. rosea* and by one third with *T. atrobrunneum*. These results agree with previous reports highlighting the antagonistic effect of *M. circinelloides* on the feces of animals passing eggs of different roundworms. In the pig manure sprayed *M. circinelloides* spores, the viability of eggs of roundworms reduced by 53% [20]. By spraying spores of *M. circinelloides* or *Verticillium* sp. on feces of captive lynxes, the viability of eggs of *T. leonina* decreased by 58% and 67%, respectively [21]. Fifty percent of the eggs of *Trichuris* sp. became non-viable by 30 days after the exposure to either *M. circinelloides* or *T. atrobrunneum*. Nevertheless, to the best of our knowledge, the control of Strongylids has been focused on trapping the larvae originating from the eggs by using nematophagous trapping species [22], so no information is available for the effects of ovicide fungi on the eggs of these nematodes.

Infection by roundworms relies on the accidental ingestion of eggs with a second-stage larva (L2) inside; hence, it would be very helpful to delay their development in the soil. Moreover, the oral ingestion of STH infective stages is not the only method of human infection, and it is important to note that the L3 larvae of the canine hookworm *A. caninum* or *A. brazilienze* are responsible for the cutaneous larva migrans, which affects tourists enjoying beaches [23]. In the present research, the Soil Contamination Index has been counseled for trying to improve the understanding of the risk of these pathogens, and it was demonstrated that one application of spores of ovicide fungi can drop the levels of soil contamination by helminths by one third–four fifths, which is especially interesting for the Strongylids due to the lack of previous investigations. Thirty days after spraying spores of *M. circinelloides* directly on feces of captive lynxes, 32% of the eggs of *T. leonina* reached the infective stage (L2), and 29% in the presence of *Verticillium* sp., while 86% did it in the unexposed controls [21].

One important issue is based on the growing reports of zoonotic parasites found in animal wastes commonly used as fertilizers on crops [24]. Despite the need for chemical fertilizers is potentially reduced, the application of raw manure increases the risk of contamination with STHs in soil and cultivated vegetables [25,26,27]. This reinforces the need to appropriate sanitation is observed, together with preventing animal infection to also avoid infection among humans. The control of STHs among pets, livestock, and captive wild animals has been supported by anthelmintic treatments during recent decades, but a decrease in the expected efficacy was detected, leading to extreme situations characterized by the emergence of strains of parasites showing a hereditary capability to resist the action of anthelmintic compounds, known as anthelmintic resistance [28,29]. In this line, targeted strategic treatments have arisen to preserve the effects of different anthelmintics by treating only individuals who certainly require such a therapy [30]. Other successful strategies are being considered, consisting of the administration of a blend of spores of *M. circinelloides* (ovicide) and *D. flagrans* (larvicide) as food formulations. Effective results have been obtained against the infection by roundworms and Strongylids after spraying the blend of spores directly on nutritional pelleted feed before provided to puppies [17], or by feeding domestic and captive herbivores with pellets industrially manufactured with the blend, which also constitutes a strategy very easy to observe without adding extra tasks to farmers or animal keepers [31,32,33].

The control of STHs in humans fundamentally relies on improving sanitation and the administration of anthelmintics, frequently as preventive therapy in low-and middle-income countries. In spite of satisfactory results are obtained, some drugs have been shown to be unable to eliminate infections or prevent reinfections [34,35,36]. Therefore, fungi could be used as an additional tool as part of a multidisciplinary response by considering a One Health approach to promote soil health and, as a result, human and animal health. Accordingly, as demonstrated among livestock species, the frequency of deworming could be lessened, which contributes to maintain the effectiveness of parasiticide drugs and to avoid the appearance of anthelmintic resistance [28,29,30]. Based on the spores of *C. rosea* and *T. atrobrunneum* that were obtained in a submerged culture, direct spraying on human stools and/or the ground could be helpful to limit the presence of STHs—not only in developing countries but also in gardens, sandpits, or recreational areas where children might take-in the infective stages of parasites that develop in the soil from feces passed by animals (both domestic and wild species), but additional research is required to demonstrate this phenomenon.

The findings of the present investigation have been obtained after only one spraying of spores directly on the feces of parasitized animals, placed in plastic boxes to allow that eggs of the parasites could be recovered and examined. The results collected point a high usefulness of this strategy for limiting contamination by helminths in the soil, but further investigations are in progress to ascertain the beneficial effect of frequent/multiple applications of spores, together with the examination on soil samples, to get more accurate knowledge of the antagonistic effect on the pathogens.

## 4. Materials and Methods

### 4.1. Study Design

The current investigation comprised two experiments. First, the helpfulness to reduce the risk of infection by *A. suum* was assayed by adding filamentous fungi onto the feces of pigs. The second experiment consisted of trying to lessen the risk of infection by third-stage larvae (L3) of nematodes. In this case, eggs of *Lemurostrongylus* sp. were utilized because this species develops an external phase in the soil identical to that of hookworms (*Ancylostoma* spp.), with the first-stage larva (L1) evolving inside the egg, emerging, and molting in the soil into the L2 and L3 stages—the infective stages for lemurs (*Eulemur fulvus albifrons*).

### 4.2. Spores of SFF (Soil Filamentous Fungi)

Two strains of soil filamentous fungi (*Clonostachys rosea*, CECT21110, and *Trichoderma atrobrunneum*, CECT20999) were cultured in submerged media to obtain their spores. These strains were isolated by the COPAR Research Group (GI-2120; University of Santiago de Compostela, Lugo, Spain) and deposited at the Spanish Type Culture Collection (CECT, Valencia, Spain).

### 4.3. Soil-Transmitted Helminths (STHs)

#### 4.3.1. *Ascaris suum*

The feces of 16 pigs reared in a private care farm (Monforte de Lemos, Lugo, Spain; 42°46′22.218″ N; 7°52′27.153″ W) were collected directly from the rectum and analyzed by means of a coprological flotation test [21,37]. Briefly, 4 g feces of each sample were emulsified in 41 mL water, filtered through a 150 µm mesh and two 12 mL filled tubes, and centrifuged at 1500 rpm for 10 min. After discarding the supernatant, 10 mL saturated sodium chloride solution (gravity = 1.2) was added to the sediment and then observed in a McMaster chamber under a light microscope (Leica DM2500) at 10×.

Because the eggs of the roundworm *A. suum* were identified, on the next day, three samples were collected again from each pig, and all were homogenized carefully by means of a tissue grinder. Once all the samples were properly mixed, three portions were prepared. Eight samples (4 g each) were taken from every portion of feces to establish the initial parasitic burden by means of the flotation test and then 16 boxes were filled with 4 g feces in each portion (total: 48 boxes):

Control: boxes provided with 2 mL of water.
CR: sprayed with 2 mL containing 2 × 10^5^ spores of *C. rosea*.
TA: sprayed with 2 mL of 4 × 10^5^ spores of *T. atrobrunneum*.

To mimic the environmental conditions as closely as possible, and also taking into account that the feces could be collected for further analysis, one hole (1 mm diameter) was made on the top of each wall of polypropylene translucent boxes (15 × 6 × 15 cm) (1.3 L volume) to allow aeration and because the feces were at a similar humidity and temperature to the values outside. Then, we covered the boxes with polypropylene lids to avoid the disintegration of the feces by rain, insects, or animals.

#### 4.3.2. *Lemurostrongylus* sp.

Feces of white-fronted lemurs (*Eulemur fulvus albifrons*) were collected from the ground of a parcel in the Marcelle Zoological Park (Outeiro de Rei, Lugo, Spain; 43°4′14.71″ N, 7°37′53.50″ W) and analyzed using a flotation test to confirm the previous findings for eggs of the strongylid nematode *Lemurostrongylus* sp. Two days later, the feces were taken again and processed as explained before to prepare three portions. After estimating the initial counts of the eggs (by analyzing eight samples of 4 g from every portion through a flotation test), 15 boxes were prepared for each portion and filled with 4 g (total: 45 boxes):

Control: boxes provided with 2 mL of water.
CR: sprayed with 2 mL containing 2 × 10^5^ spores of *C. rosea*.
TA: sprayed with 2 mL of 4 × 10^5^ spores of *T. atrobrunneum*.

### 4.4. Analysis of the Effect of SSF on the Eggs of STHs

All the boxes were positioned in a grassland under 8–17 °C and 65–77% relative humidity. According to previous investigations [21,37], four boxes with the feces of pigs in each group were taken weekly during a period of four weeks, and once in the lab, the fecal content (without being weighed) was analyzed by a flotation test to estimate the counts of EPG. Then, the percentage of the recovery rate was calculated weekly for each group:

% RR (recovery rate) = (EPG_weekX_/EPG_week0_) × 100.

To assess the viability of the eggs, the developmental rate, and the possible damage caused by the SSF to the eggs, 100 µL aliquots were collected from the upper fraction of the tubes and placed between glass slides and coverslips for observation under a light microscope at 10× and 20× until a minimum of 100 eggs was observed [21]. This procedure was repeated two times for each sample.

The eggs of *A. suum* were sorted into a permanently unviable group based on a loss of shell continuity, contraction or rupture, the observation of vacuolizated cytoplasm, and a lack of larval movement after light stimulation [38]. The viable eggs of roundworms were classified into zygotes (no cellular division), eggs with developing embryos (from 1 cell to gastrula), L1 (first stage larva), and L2 (infective stage).

Because the first stage larvae of Strongylids emerged starting at 6–10 days, the boxes containing the feces of lemurs were maintained in a grassland for 21 days. Five boxes of every group were taken weekly and analyzed in the lab as previously described. The evaluation of the SSF activity on the eggs of *Lemurostrongylus* sp. also involved identifying the eggs as irreversibly non-viable or viable. The eggs were subsequently classified as zygotes, eggs with developing embryos, and L1.

### 4.5. Reduction of Soil Contamination by STHs

To ascertain the effect obtained by spraying fungal spores onto the feces of pigs and lemurs, two parameters were calculated weekly, the Viability Reduction (VR):

VR*_Ascaris suum_* (%) = [(100 − % Viable Eggs_WeekX_)/% Viable EPG_Week0_] × 100

VR*_Lemurostrongylus_*_sp._ (%) = [(100 − % Viable EPG_WeekX_)/% Viable EPG _Week0_] × 100

With the aim to facilitate the understanding of the results and to establish a direct correlation with the risk of contamination by helminths in the soil, the Soil Contamination Index (SCI) was defined as:

SCI*_A. suum_* (%) = (% Viable Eggs × % of Eggs with L2)/100

SCI*_Lemurostrongylus_*_sp._ (%) = (% Viable Eggs × % of Eggs with L1)/100.

It is interesting to note that high SCI values indicate a high risk of causing infection.

### 4.6. Statistical Analyses

Using the Kolmogorov–Smirnov probe, the data collected for the fecal egg counts were found not to be normally distributed (Z = 2.870, *p* = 0.001), and the Levene probe showed that the variances were not homogeneous (Statistic = 22.003, *p* = 0.001). Thus, a nonparametric Mann–Whitney U test was performed (significance level *p* < 0.05). All the probes were employed using the statistical software SPSS, version 21 (IBM SPSS, Inc., Chicago, IL, USA).

## 5. Conclusions

The data collected in the present study confirm the usefulness of spreading spores of the filamentous fungi *C. rosea* and *T. atrobrunneum* directly onto the feces of animal species to reduce the viability of eggs of zoonotic STHs, together with the possibility of attaining the infective stages in the soil. This provides a sustainable approach under a One Health context to reduce the risk of zoonotic STHs in humans.

## Figures and Tables

**Table 1:** Analysis of the eggs of *A. suum* in the feces of pigs. Control: not exposed to fungi; CR: exposed to the parasiticide fungus *Clonostachys rosea*; TA: exposed to *Trichoderma atrobrunneum*; EPG: eggs per gram of feces; RR: recovery rate.

Week	Treatment	*Ascaris suum*		*Lemurostrongylus* sp.	
		EPG	RR	EPG	RR
0	Control	563 ± 256		490 ± 188	
	CR	625 ± 290		750 ± 177	
	TA	525 ± 155		670 ± 303	
1	Control	475 ± 240	(84%)	400 ± 187	(81%)
	CR	475 ± 290	(76%)	530 ± 164	(70%)
	TA	363 ± 95	(69%)	440 ± 207	(65%)
2	Control	413 ± 229	(73%)	325 ± 155	(67%)
	CR	350 ± 178	(56%)	420 ± 196	(54%)
	TA	275 ± 65	(52%)	340 ± 167	(50%)
3	Control	313 ± 131	(56%)		
	CR	313 ± 189	(50%)	320 ± 164	(41%)
	TA	225 ± 50	(43%)	270 ± 84	(43%)
4	Control	288 ± 193	(51%)		
	CR	263 ± 131	(42%)		
	TA	200 ± 41	(38%)		

**Table 2:** Analysis of the eggs of *Ascaris suum* in the feces of pigs. Control: not exposed to fungi; CR: exposed to the parasiticide fungus *Clonostachys rosea*; TA: exposed to *Trichoderma atrobrunneum*; DE: developing embryos; L1: eggs containing a first-stage larva; L2: eggs containing a second-stage larva.

Week	Treatment	Viable				Non-Viable
		Zygote	DE	L1	L2	
0	Control	127 ± 11 (100%)	0 (0%)	0 (0%)	0 (0%)	1 ± 1
	CR	121 ± 9 (100%)	0 (0%)	0 (0%)	0 (0%)	1 ± 1
	TA	119 ± 3 (100%)	0 (0%)	0 (0%)	0 (0%)	1 ± 1
1	Control	34 ± 3 (29%)	85 ± 13 (71%)	0 (0%)	0 (0%)	6 ± 3
	CR	33 ± 3 (37%)	55 ± 9 (63%)	0 (0%)	0 (0%)	37 ± 8
	TA	52 ± 5 (51%)	49 ± 4 (49%)	0 (0%)	0 (0%)	35 ± 5
2	Control	7 ± 10 (7%)	24 ± 4 (22%)	77 ± 5 (70%)	1 ± 2 (1%)	12 ± 7
	CR	15 ± 4 (21%)	21 ± 6 (29%)	35 ± 8 (48%)	1 ± 2 (2%)	58 ± 24
	TA	32 ± 5 (39%)	34 ± 16 (40%)	16 ± 1 (20%)	1 ± 1 (1%)	56 ± 18
3	Control	2 ± 1 (2%)	11 ± 3 (10%)	32 ± 3 (29%)	66 ± 5 (59%)	25 ± 10
	CR	7 ± 4 (13%)	11 ± 4 (20%)	19 ± 5 (36%)	17 ± 5 (31%)	68 ± 13
	TA	13 ± 3 (27%)	14 ± 6 (28%)	12 ± 3 (24%)	10 ± 2 (21%)	84 ± 7
4	Control	1 ± 1 (0.5%)	3 ± 2 (2%)	13 ± 5 (12.5%)	88 ± 4 (85%)	28 ± 6
	CR	4 ± 1 (10) (10%)	3 ± 2 (6%)	10 ± 6 (25%)	24 ± 2 (59%)	77 ± 17
	TA	4 ± 2 (12%)	6 ± 1 (19%)	10 ± 2 (30%)	13 ± 1 (39%)	84 ± 16

**Table 3:** Analysis of the eggs of *Lemurostrongylus* sp. in the feces of lemurs. Control: not exposed to fungi; CR: exposed to the parasiticide fungus *Clonostachys rosea*; TA: exposed to *Trichoderma atrobrunneum*; RR: recovery rate; DE: developing embryos; L1: eggs containing a first-stage larva.

Week	Treatment	Viable			Non-Viable
		Zygote	DE	L1	
0	Control	142 ± 10 (91%)	9 ± 4 (6%)	5 ± 1 (3%)	2 ± 1
	CR	142 ± 10 (91%)	9 ± 4 (6%)	5 ± 1 (3%)	2 ± 1
	TA	142 ± 10 (91%)	9 ± 4 (6%)	5 ± 1 (3%)	1 ± 1
1	Control	9 ± 2 (6%)	27 ± 6 (17%)	121 ± 17 (77%)	11 ± 6
	CR	38 ± 10 (26%)	43 ± 11 (29%)	66 ± 27 (45%)	18 ± 9
	TA	60 ± 15 (40%)	50 ± 10 (34%)	39 ± 9 (26%)	17 ± 7
2	Control	4 ± 1 (3%)	11 ± 2 (7%)	136 ± 8 (90%)	17 ± 5
	CR	23 ± 4 (20%)	29 ± 6 (24%)	67 ± 13 (56%)	48 ± 8
	TA	28 ± 2 (26)	25 ± 8 (23%)	56 ± 6 (51%)	46 ± 7
3	Control				24 ± 9
	CR	13 ± 3 (13%)	16 ± 8 (16%)	70 ± 5 (71%)	52 ± 8
	TA	17 ± 3 (17%)	26 ± 7 (26%)	58 ± 10 (57%)	60 ± 5
